# Panic Disorder: Is the PAG Involved?

**DOI:** 10.1155/2009/108135

**Published:** 2009-03-10

**Authors:** Cristina Marta Del-Ben, Frederico Guilherme Graeff

**Affiliations:** Psychiatry Division, Faculty of Medicine of Ribeirão Preto, University of São Paulo, Avenida Bandeirantes, 3900, 14048-900 Ribeirão Preto, SP, Brazil

## Abstract

Data from studies with humans have suggested that abnormalities of midbrain structures, including the periaqueductal gray matter (PAG), could be involved in the neurobiology of panic disorder (PD). The electrical stimulation of the PAG in neurosurgical patients induces panic-like symptoms and the effect of drugs that are effective in the treatment of PD in the simulation of public speaking model of anxiety is in agreement with data from animal models of PD. Structural neuroimaging studies have shown increases in gray matter volume of midbrain and pons of PD patients. There is also evidence of lower serotonin transporter and receptor binding, and increases of metabolism in the midbrain of PD patients. Nevertheless, these midbrain abnormalities can not be considered as specific findings, since neuroimaging data indicate that PD patients have abnormalities in other brain structures that process fear and anxiety.

## 1. Introduction

Panic disorder (PD) is a common and incapacitating mental disorder characterized by the recurrence of spontaneous panic attacks, followed by a persistent concern about having additional attacks, worry about the implications of the attack or its consequences, and a significant change in the behavior related to the attacks. A panic attack is characterized as a discrete period of intense fear or discomfort, in which several symptoms, such as palpitations, pounding heart, or accelerated heart rate; sweating; trembling or shaking; sensations of shortness of breath or smothering; feeling of choking; chest pain or discomfort; nausea or abdominal distress; feeling dizzy, unsteady, lightheaded, or faint; derealization or depersonalization; fear of losing control or going crazy; fear of dying; paresthesias; chills or hot flushes, develop abruptly and reach a peak within 10 minutes. The symptoms are not related to substance abuse or general medical condition and are associated with a significant impairment of global functioning. Around 2/3 of patients with PD will also develop agoraphobia, which is defined as an anxiety about being in places or situations from which escape might be difficult, or embarrassing; or in which help may not be available in the event of an unexpected or situationally predisposed panic attack. Agoraphobic fears typically involve characteristic clusters of situations that include being outside the home alone, being in a crowd or standing in a line, being on a bridge, and traveling in a bus, train, or automobile [1].

Several brain structures that organize defensive reactions and represent the neural substrate of fear and anxiety have been implicated in the functional neuroanatomy of PD. Among those are prefrontal regions, amygdala, hippocampus, and parahippocampal area, hypothalamus, thalamus, and the periaqueductal grey matter (PAG) (for a recent review; see [2]). In regard to the latter region, animal studies have shown that electrical and chemical stimulations of the PAG cause urgent defensive reactions, such as freezing, fight, or flight. The same responses occur when the animal is faced by a clear and near threat, for instance, a predator [3]. Therefore, the PAG has been implicated in the defensive reaction to proximal threats, and drugs that increase the serotonergic function and are effective in the treatment of PD are able to reduce behaviors normally observed with the stimulation of the PAG (reviewed in [4]). Although other neurotransmitters, such as cholecystokinin [5] and glutamate [6], also appear to regulate fear/panic-related defensive behavior, the main focus of this review will be on serotonin (5-HT) since this is the main neurotransmitter affected by the drugs clinically used for the treatment of PD.

Even though the evidence that supports the involvement of the neurocircuitry underlying defensive reactions in normal and pathological fear and anxiety has mainly been obtained with preclinical research, data from studies with humans also give support to the concept that structural and functional abnormalities in midbrain structures, such as the PAG, could be involved in the neurobiology of PD. Several reviews (e.g., [2]) have brought together animal findings showing the role of PAG in fear reactions and defensive behavior to proximal threats, but data coming from studies with human beings have not been completely explored. Therefore, the focus of this review is on results from human studies, including healthy volunteers and patients with PD, which provide evidence for a participation of the PAG in the pathophysiology of PD.

## 2. Symptomatic Homology

A pivotal evidence for the involvement of midbrain structures in PD came from the induction of panic-like symptoms by electrical stimulation of the PAG in neurosurgical patients. Awaked patients submitted to the stimulation of the PAG report feelings of terror or impending death, desire to flee, palpitation, and respiratory arrest or hyperventilation [7–9]. The remarkable similarities between the effects of PAG electrical stimulation in neurosurgical patients reported above and the symptoms that occur during a panic attack led the Brazilian psychiatrist Valentil Gentil to suggest a participation of the PAG in the neurobiology of panic attacks. Commenting on the changing in the behavior of rats due to the stimulation of the dorsal PAG, Gentil remarked, “I believe that (this animal) model is particularly useful for the understanding of the pathophysiology of panic attacks, especially the “spontaneous” attacks. … (Bearing in mind that) the panic attack is a very primitive behavior … the isomorphic validity of the central gray's (PAG) poorly organized responses to *γ*-aminobutyric acid (GABA-A) antagonists and electrical stimulation to the maladaptive flight behavior of full-blown panic seems high” [10]. Further, the phenomenological resemblance between panic attacks and the effects of the electrical stimulation of the PAG in both humans and animals has been systematically explored [11, 12], the main results being summarized in Table 1.

Although the similarity between the symptoms of a spontaneous panic attack and the effects of electrical stimulation of PAG is often cited as a face-validity criterion for implicating the PAG in PD, information obtained from awaked patients about the subjective and somatic responses provoked by stimulation of PAG is rare in the recent literature. An exception is the work carried out by Green et al. [13], using deep brain stimulation, who have obtained results similar to those reported by Nashold et al. [7], four decades ago. In the procedure of deep brain stimulation, electrodes are implanted permanently into specific areas of the brain. The electrodes are connected by wires under the skin to a generator allowing continuous electrical stimulation of specific brain areas. In this study, patients had electrodes implanted inside the PAG to control neuropathic pain. It has been observed that electrodes placed more dorsally in the PAG increased systolic and diastolic arterial blood pressure, what did not occur with electrodes placed more ventrally in the PAG. Moreover, two patients with dorsal electrodes reported nausea, sweating, and anxiety, symptoms commonly observed during a spontaneous panic attack. Although this issue has not been completely established, there is some evidence pointing to an association between PD and hypertension. In this regard, it has been proposed that both conditions would share a dysfunction of brainstem structures that regulate the autonomic nervous system and are inhibited by 5-HT [14].

## 3. Experimental Anxiety in Humans

Aiming to conciliate seemingly conflicting results derived from animal studies about the role of 5-HT in anxiety, it has been proposed that 5-HT projections from the dorsal raphe nucleus (DRN) facilitate inhibitory avoidance in limbic forebrain structures, predominantly amygdala and frontal cortex, while inhibit escape in the dorsal PAG [12]. This arrangement may have adaptive value, since it allows inhibition of fight/flight behavior in situations where threat is only potential or remote.

**Figure 1:**
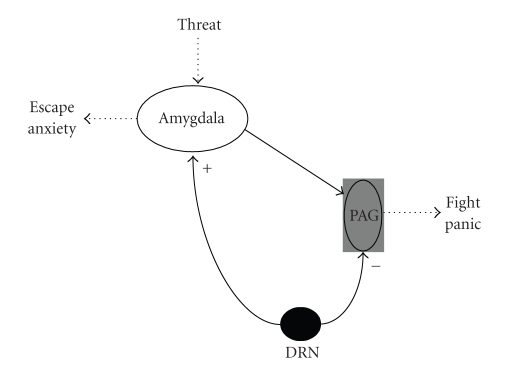
Schematic representation of the dual role of serotonin on fear and anxiety, according to Deakin and Graeff theory.

More recently, Lowry et al. [15] have shown that the 5-HT projections to cortical and limbic structures arise from a neuronal set located in a specific part of the caudal DRN, which is particularly sensitive to stressful stimuli. The rostral projections from these neurons seem to constitute a mesocorticolimbic 5-HT system that modulates defense. Based on correlations between the pharmacological efficacy of antidepressants and anxiolytic drugs in anxiety disorders and the results obtained in experimental models of anxiety in humans (discussed below), it has been further suggested that generalized anxiety disorder (GAD) would be related to the inhibitory avoidance and conditioned anxiety, whereas PD would be related to the escape response and innate fear [16]. A schematic representation of the hypothesis on the dual role of 5-HT in anxiety and defense is represented in Figure 1.

This theoretical model has been systematically tested using two experimental procedures that generate anxiety in human beings: the simulated public speaking (SPS) and the skin conductance response (CSCR) tests (for a review; see [17]). It is important to note that this experimental approach is different from that used in pharmacological challenges aimed at provoking a panic attack in vulnerable individuals. In this case, the most used are the infusion of sodium lactate and the inhalation of CO_2_. Both challenges induce panic attacks in around 60 to 80% of panic patients, as compared to 0 to 20% of healthy controls. This seems to be a very specific response, since these challenges do not cause panic attacks in phobic or obsessive compulsive patients. Moreover, pharmacological studies have evidenced that antidepressant treatment decreases the vulnerability of panic patients to lactate and/or CO_2_ [18]. The similarities between the effects of lactate and CO_2_ led to the hypothesis that both challenges have a common mechanism of action, causing an intraneuronal hypercapnia in brain areas that are stimulated by CO_2_ during suffocation. The sensitivity of such suffocation alarm system would be abnormally heightened in PD patients [19].

Basically, the SPS test consists in the preparation and performance of a speech in front of a videocamera, with the participant seeing his/her own image on a TV screen. Subjective and physiologic measures of anxiety are taken before, during and after the speech. The emotional state induced by SPS is supposed to be species-specific fear, given that fear of speaking is highly prevalent in the general population [20] and occurs in healthy persons, irrespective of their personality trait to react with more or less anxiety to stressful situations [21]. Pharmacological studies have shown that drugs that facilitate 5-HT function decrease, whereas drugs that impair 5-HT function increase speaking fear [17]. On the other hand, the CSCR test is based on classical conditioning theory, consisting in the presentation of 10 neutral tones (habituation phase), followed by a neutral tone paired with a loud white noise (acquisition phase) and by the representation of 10 neutral tones (extinction phase). During the procedures, measures of skin conductance are taken. Drugs that increase 5-HT tend to facilitate conditioning [17].

Several 5-HT acting drugs have been assayed in these tests. For instance, a single dose of chlomipramine [22] and nefazodone [23] administered to healthy volunteers increased the fear provoked by the SPS, and this effect has been related to the clinical worsening observed at the beginning of the treatment with antidepressants [24–26]. While some animal studies have shown an increase in cortical extracellular level of 5-HT following acute administration of antidepressants [27–29], others have shown a greater increase of extracellular 5-HT in the raphe nuclei than in the neocortex [30]. If so, a single dose of an antidepressant would preferentially increase the concentration of 5-HT near the cell bodies of serotonergic neurons, which would activate somatodendritic 5-HT_1A_ autoreceptors, reducing neuronal firing [31] and, consequently, leading to a decrease in the release of 5-HT in the synaptic cleft. Therefore, the fear-enhancing effect of a single dose of antidepressants in SPS could be due to a lack of 5-HT inhibition of brain systems that generate panic attacks, likely to be localized in the dorsal PAG [4, 32].

In agreement with the hypothesis about the dual role of 5-HT in fear and anxiety, ritanserin, a 5-HT receptor antagonist, has shown opposite effects in the SPS and CSCR tests, prolonging the fear induced by SPS and decreasing conditioned skin conductance responses [33]. These results resemble reported clinical results with ritanserin, showing improvement of GAD [34], but a tendency to aggravate PD [35, 36]. To the opposite direction, the 5-HT releaser d-fenfluramine has been shown to reduce SPS-induced fear [37] and to improve PD [38, 39]. In contrast, d-fenfluramine tended to increase the amplitude of conditioned skin conductance responses, suggesting an anxiogenic-like effect [37].

Hence these pharmacological results with experimentally-induced fear and anxiety in humans are in agreement with the hypothesis that 5-HT enhances anxiety, which can be evaluated by the CSCR test, whereas inhibits fear, which can be assessed by the SPS test. The former effect would be related to the action of 5-HT on forebrain structures and the latter to its action on dorsal PAG. It has been well demonstrated that the chronic use of drugs that increase the availability of serotonin in the synaptic cleft is effective for the treatment of PD [40] and it has been proposed that the reduction in the occurrence of panic attacks with the use of antidepressants could be due to enhancement of the inhibitory action of serotonin on the PAG [4].

## 4. Panic Patients and Experimental Models of Anxiety

It is important to note that the SPS is not taken as a model of panic attack and it is not expected to provoke panic attacks in susceptible individuals. The possible association between the experimental model and the mental disorder is based on the rationale that public speaking would engage the neural substrates involved in the process of innate fear, which would be abnormal in PD.

Therefore, if the predictions derived from pharmacological studies with the human tests discussed above are correct, it would be expected that patients with the diagnosis of PD and healthy volunteers would perform differently in the SPS, but not in the CSCR test, given that the former would engage the brain mechanisms implicated in the neurobiology of PD, but the latter would not.

Aiming to test this hypothesis, we submitted panic patients free of treatment to both models of anxiety [41]. As predicted, controls and panic patients showed a similar response to CSCR. In contrast, during the SPS test, panic patients demonstrated higher levels of subjective anxiety than healthy volunteers from the beginning to the end of the experimental session but were less responsive to the speaking challenge. The profile of the subjective response of panic patients to the SPS test bears a resemblance to the effect of metergoline, a nonselective 5-HT-receptor blocker, given to healthy volunteers. Metergoline enhanced the subjective anxiety before and after the speech, but not during the preparation or the performance of the speech [42]. These results were in agreement with the suggestion that an impairment of the 5-HT function leading to a reduced of the inhibition of PAG may be present in the neurobiology of PD [16].

Using a similar protocol [43, 44], new groups of symptomatic panic patients and healthy controls were submitted to the SPS test. In addition, a third experimental group composed by panic patients who had become nonsymptomatic after long-term pharmacological treatment with antidepressant drugs was added. The aim was to verify whether the differences between healthy subjects and PD patients, if replicated, would remain after recovery, being thus related to a vulnerability trait, or otherwise decrease, and therefore being related to the clinical condition (state).

**Figure 2:**
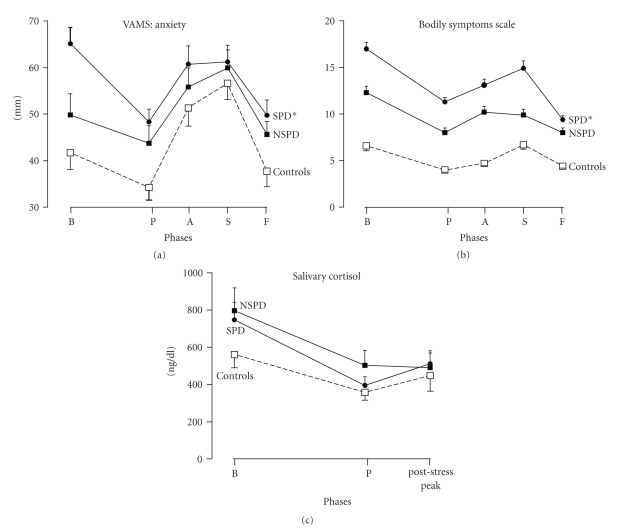
Changes in anxiety (VAMS, upper panel), bodily symptoms (BSS, mild panel), and salivary cortisol levels (lower panel) induced by simulated public speaking (SPS) in symptomatic panic patients (SPD), nonsymptomatic patients (NSPD), and healthy controls. The phases of the experimental session are beginning (B), pretest (P), anxiety during speech preparation (A), performance anxiety during the speech (S), and final (F). Points in the curves indicate mean values and vertical bars the S.E.M. Figure modified from Garcia-Leal et al. [43] and Parente et al. [44]. * = significant difference (*P* < .05) from controls.

As can be seen in Figure 2, and in agreement with the former study, symptomatic drug free panic patients had more subjective anxiety during the experimental session than controls, despite the changes introduced in the procedures to minimize differences in expectancy and familiarity that might enhance or decrease initial anxiety, respectively. A more prolonged period of habituation decreased the anxiety in all groups, but the response to the SPS challenge was smaller in symptomatic patients than in normal controls. Moreover, nonsymptomatic patients stand between controls (below) and symptomatic panic patients (above) with regard to subjective anxiety, measured by the visual analogue mood scale (VAMS) and to bodily symptoms, measured by the total score of the bodily symptoms scale (BSS). Therefore, these measures seem to be related to the magnitude of clinical manifestations of PD rather than to a vulnerability trait, since they were affected by pharmacological treatment.

This study has also shown a significant decrease in the level of salivary cortisol from the initial to the pretest phases of the experimental session, in parallel with habituation of the anticipatory anxiety induced by the experimental setting. Additionally, a positive correlation between levels of subjective anxiety and of salivary cortisol has been found in control subjects at the initial phase of the experimental session. In contrast, salivary cortisol did not increase during the 60 minutes following the end of the speech, neither in patients, nor in controls, despite the levels of anxiety measured during speech preparation and performance being at least as high as those at the onset of the experimental session. Therefore, the SPS task does not seem to increase cortisol secretion. In agreement with these results, neither spontaneous panic attacks [45] nor the electrical stimulation of the dorsal PAG of the rat [46] activates the hypothalamic-pituitary-adrenal axis.

A final remark about the possible abnormal processing of innate fear in PD has come from a study carried out in our laboratory with patients with social anxiety disorder (SAD) submitted to the SPS test (MC Freitas, A Santos Filho, F Osório, SR Loureiro, CM Del-Ben, AW Zuardi, FG Graeff, JAS Crippa, unpublished results). SAD and PD are different anxiety disorders, but they keep some similarities, such as the response to the treatment with antidepressants that act on 5-HT function. However, in comparison to healthy controls, SAD patients have shown a larger enhancement of the fear induced by the SPS, what is different from the results obtained with PD patients. For that reason, we could speculate that the lower fear response induced by SPS could be specific to PD and related to abnormal functioning of brain structures involved in the process of innate fear.

## 5. Neuroimaging Data

As discussed earlier, evidence from preclinical studies suggests that the neural substrates involved in the defensive reactions to environmental threats of mammalian species could be implicated in the pathophysiology of PD. The main brain structures possibly involved in the neurobiology of PD encompass the prefrontal cortex, anterior cingulated cortex, hypothalamus, amygdala, hippocampus, and the midbrain, including the periaqueductal grey matter [2].

Structural neuroimaging studies, using magnetic resonance imaging (MRI), have shown that anatomical brain abnormalities, particularly in the temporal lobes, are more frequently observed in panic patients than in controls [47–49]. A quantitative evaluation of specific brain structures has also demonstrated differences between PD patients and healthy volunteers, characterized by a reduction of the volume of temporal lobes, amygdala, and hippocampus (trend) in PD patients compared to controls [50–52].

Voxel-based morphometry (VBM) is a more sophisticated approach of structural neuroimaging that provides an automated method of segmentation into gray matter, white matter, and cerebrospinal fluid (CSF) compartments and allows the investigation of differences in regional volumes along the whole brain [53]. Using the VBM technique, Protopopescu et al. [54] have shown an increase in gray matter volume of the midbrain and rostral pons of the brainstem of panic patients compared with healthy controls. At a lower significance threshold, they have also reported increased ventral hippocampal and decreased regional prefrontal cortex volumes in PD.

**Figure 3:**
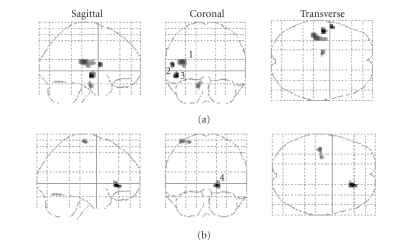
Statistical parametric maps displaying significant differences in gray matter volume of PD patients (*n* = 19) relative to healthy controls (*n* = 20). (a) Increased gray matter volume, (b) decreased gray matter volume. 1 = left insula; 2 = left superior temporal gyrus; 3 = midbrain; 4 = right anterior cingulate.

In a recently published study [55], we have also found a relative increase in gray matter volume of midbrain and pons (on left) in panic patients. As it can be seen in the Figure 3, additional findings include increase in gray matter volume of the left insula and left superior temporal gyrus and a relative gray matter decrease in the right anterior cingulate cortex. The anterior insula has close connections to the amygdala and, together with the ventromedial prefrontal cortex, anterior cingulate cortex, hypothalamus, and periaqueductal gray matter, is considered as part of a network that modulates the identification of, and the response to, aversive or threatening stimuli [56] and has been proposed as a key structure involved in the neurobiology of anxiety disorders [57]. In particular, the increase of gray matter volume of midbrain is in agreement with the proposition that periaqueductal gray matter would be implicated in the pathophysiology of PD as well in the antipanic action of antidepressant drugs [2, 4, 16].

Functional neuroimaging studies have also contributed to a deeper understanding of the neural substrates of PD. In a seminal work, using positron emission tomography (PET), Reiman et al. [58] have found abnormalities in the parahippocampal gyri, characterized by an abnormal asymmetry (left less than right) of the regional cerebral blood flow (rCBF), observed, during rest, in panic patients vulnerable to the lactate challenge. Further functional studies have also shown alterations in the metabolism or blood flow of hippocampus and parahippocampal areas of panic patients [59–65] and this seems to be the most consistent finding across the studies with functional neuroimaging in PD. Other areas implicated in the pathology of PD by functional studies are prefrontal cortex [59, 60, 65], anterior cingulate gyrus [62, 65], superior temporal cortex [61, 62], amygdala [63, 64], hypothalamus [62], and thalamus [63, 64].

Considering that the PAG is a small brain structure, the detection of dysfunctions of its metabolism is not straightforward, due to limitations of the neuroimaging technique itself. Even so, some studies have reported abnormalities in the midbrain of panic patients.

Just before being submitted to a pentagastrin challenge, panic patients, compared with healthy volunteers, have shown an increase of blood flow in parahippocampal gyrus, left hippocampus, right temporal lobe, orbitofrontal cortex, anterior cingulate gyrus, hypothalamus, thalamus, and midbrain, “probably” PAG [62]. Interestingly, bilateral insula, inferior frontal gyrus, and right amygdala have shown abnormalities in their metabolism in the opposite direction, with a decrease of blood flow, what suggests that the inhibitory function of forebrain structures over phylogenetically more primitive structures, such as the PAG, would be impaired in panic patients.

In the same direction, Sakai et al. [63, 64] have found higher levels of glucose uptake in the midbrain, caudal pons, and medulla in panic patients than controls. They also have shown an increase of the metabolism in bilateral amygdala, hippocampus, and thalamus. In a further study, the same group [65] has shown a decrease of glucose uptake in the right hippocampus, left anterior cingulate, left cerebellum, and pons and an increase of glucose uptake in bilateral medial prefrontal cortices in panic patients that had shown clinical improvement after a cognitive-behavioral therapy intervention. These changes in the brain metabolism with the treatment with antidepressants or cognitive-behavioral therapy have not been found in previous studies [60, 66]. More interestingly for this review is the fact that they have demonstrated a correlation between the percent changes in glucose utilization in the midbrain “around PAG” and those of the number of panic attacks during the 4-week period before each scan, which shows a direct relation between PAG metabolism and the occurrence of panic attacks.

As discussed above, serotonin has been largely implicated in the pathophysiology of panic disorder, and some evidence from neuroimaging studies suggests alterations in the 5-HT system of PD patients. The intravenous administration of d-fenfluramine, which induces the neuronal release of serotonin, has provoked a decrease of blood flow in the left posterior parietal-superior temporal cortex in panic patients [66]. A lower volume of distribution of a selective radioligand of serotonergic receptors 5-TH_1A_ type has been described in the anterior cingulate, posterior cingulate, and raphe of nonmedicated panic patients relative to controls [67]. A significant decrease in the serotonin transporter (5-HTT) binding in the midbrain, temporal lobes, and thalamus of symptomatic panic patients free of medication has also been reported [68]. However, in comparison to patients with current symptoms, panic patients in remission and free of medication have normal 5-HTT binding properties in the midbrain and in the temporal regions but still show significantly lower thalamic 5-HTT binding. Considering all the patients' (current and in remission) significant negative correlations between the severity of panic symptoms and the midbrain, temporal lobe, but not thalamic 5-HTT binding, has also been demonstrated [68].

These abnormalities in the binding of 5-HT receptors and transporter in midbrain areas are in agreement with the hypothesis that the occurrence of panic attacks would be caused by spontaneous activations of the fight/flight response organized by the PAG and inhibited by 5-HT [16].

Although few studies have applied functional magnetic resonance imaging (fMRI) in panic patients so far, most of them confirmed alterations in the brain areas previously supposed to be involved in the neurocircuitary of PD. In a paradigm of mental imagery of neutral, moderate, and high anxiety situations, panic patients have shown increased neuronal activation in the inferior frontal cortex, hippocampus, and anterior and posterior cingulate, extending into the orbitofrontal cortex bilaterally, during the anxious blocks compared to neutral blocks [69]. Panic patients have also shown significantly higher activation in left posterior cingulate and left middle frontal cortices and a more pronounced asymmetry (right > left) in parahippocampal regions in response to a threat-related stimuli, in comparison to healthy volunteers [70]. Compared to healthy controls, panic patients have demonstrated significantly less activation to fearful faces in the cingulate cortex and the amygdala, bilaterally [71].

For our knowledge, none of these fMRI studies have reported functional alterations in the midbrain. This not only can be due to limitations of the technique itself that do not allow the analysis of changes of fMRI signal in such a small area but also can be related to the hypotheses underlying the studies, which drive the choice of the paradigm of psychological activation and determine the regions of interest where the possible alterations will be looked for. For instance, in the light of the comprehensive view of the neurobiology of anxiety and fear proposed by Deakin in Graeff [16] a suitable paradigm to provoke enough haemodinamic response of midbrain areas would be related to the process of innate fear in humans.

In this regard, a very interesting work carried out with healthy volunteers has brought some light to this discussion. Mobbs et al. [72] have evaluated the effects on brain activation of the distance of a virtual predator. In this paradigm, participants could control the movements of a virtual prey (represented as a dot) in a labyrinth presented on a video-screen, using a keyboard, aiming to avoid a virtual predator (represented by a triangle) with the ability to chase, capture, and inflict pain. In the case of the predator caught the prey, two levels of pain represented by either one or three electric shocks administered to one finger of the participant. When the predator was far from the prey, the haemodinamic responses observed in the prefrontal cortex and lateral amygdala were more pronounced, particularly when the expected shock intensity was low. In contrast, when the predator was closer, the haemodinamic response shifted to the central amygdala and the PAG, reaching the maximum of activation when the highest level of pain was anticipated. Even more interesting, there was a positive correlation between PAG activation and the reported subjective degree of dread and decreased confidence of escape. These results give strong support to the role of the midbrain PAG in proximal defense, and possibly panic, as early proposed [2, 14].

## 6. Conclusions

In accordance to results coming from animal research, data from experimental models of anxiety, pharmacological challenges, and neuroimaging studies carried out with healthy volunteers and patients with PD point to the involvement of the PAG in the neurobiology of PD. Nevertheless, these midbrain abnormalities cannot be considered as specific findings, since neuroimaging data have also shown that PD patients have changes in other brain structures that participate in the regulation of fear and anxiety. In a more integrative approach, it is reasonable to suppose that a dysfunction of PAG could be part of a global dysfunction that affects a network of related brain structures, or even a consequence of other dysfunctions, such as low 5-HT function, impairment of inhibitory efferent pathways from rostral brain areas, or both. Further studies conciliating biological vulnerability, environmental influences and, mainly, the connectivity among different brain structures with a clear hypothesis-driven approach are needed.

## Figures and Tables

**Table 1:** Phenomenological similarities between panic attacks and effects of electrical stimulation of the periaqueductal gray matter (PAG) in humans and rats. Adapted from Jenck et al. [11] and Schenberg et al. [12].

Spontaneous panic attack	Stimulation of dorsal PAG in humans	Stimulation of dorsal PAG in rats
Intense fear or discomfort	Panic, terror	—
—	Intense distress	Aversion
Palpitations, pounding heart, or accelerated heart rate	Tachycardia	Tachycardia
Sweating	Sweating	—
Trembling or shaking	Sensation of vibration	—
Sensations of shortness of breath or smothering	—	Tachypnea
—	Hyperventilation	Hyperventilation
—	Apnea	—
Chest pain or discomfort	Chest and heart pain	—
Nausea or abdominal distress	Bladder voiding urge	Micturation
—	—	Defecation
Fear of dying	“Scared to death”	Escape responses
Chills or hot flushes	“Burn/cold” sensations	—
